# Impact of a Virtual Three-Dimensional Thyroid Model on Patient Communication in Thyroid Surgery: A Randomized Controlled Trial

**DOI:** 10.3390/cancers18020241

**Published:** 2026-01-13

**Authors:** Zhen Cao, Qiyao Zhang, Shangcheng Yan, Zhihong Qian, Xiequn Xu, Ziwen Liu

**Affiliations:** 1 Department of General Surgery, Peking Union Medical College Hospital, Chinese Academy of Medical Sciences and Peking Union Medical College, Beijing 100730, China; 2 Department of Basic Medical Sciences, School of Medicine, Tsinghua University, Beijing 100084, China

**Keywords:** thyroid cancer, preoperative communication, three-dimensional model, patient comprehension, shared decision-making

## Abstract

Background: Effective preoperative patient counseling is essential to shared decision-making. In thyroid surgery, patient communication can be complicated by the complex anatomy and variable surgical approaches, which may not be fully conveyed through conventional verbal explanations or schematic drawings. Virtual three-dimensional (3D) thyroid models may provide an intuitive tool to enhance patient comprehension. Methods: We conducted a randomized controlled trial at Peking Union Medical College Hospital with 94 newly-diagnosed thyroid cancer patients scheduled for thyroidectomy. Participants were assigned to either the control group (*n* = 47), which received preoperative drawing-based counseling, or the intervention group (*n* = 47), which utilized a virtual 3D model for communication. The Thyroid Navigator app, developed by Kuma Hospital, was used to provide dynamic 3D representation of the thyroid gland, surrounding structures, and potential surgical procedures. After standardized preoperative consultations, patients were surveyed to assess their understanding in pertinent anatomy and postoperative complications. Results: Patients in the 3D model group demonstrated similar correct response rates in lesion localization (*p* = 0.536) or parathyroid gland recognition (*p* = 0.071), but significantly higher accuracy in identifying the recurrent laryngeal nerve and the extent of lymph node dissection compared with the control group (*p* < 0.05). Moreover, comprehension of the causes of major postoperative complications—including hoarseness (recurrent laryngeal nerve injury, *p* = 0.004), hypocalcemia (parathyroid gland impairment, *p* = 0.015), and bleeding (inadequate hemostasis, *p* = 0.008)—was significantly improved in the 3D model group. Conclusions: Use of a virtual 3D thyroid model significantly improves patient comprehension of thyroid anatomy, surgical procedures, and potential complications, thereby enhancing clinician–patient communication. Virtual 3D models represent a practical and cost-effective supplement to conventional counseling in thyroid surgery, offering clear benefits in patient education and shared decision-making.

## Simple Summary

Clear preoperative communication is essential for patients undergoing thyroid surgery, but understanding thyroid anatomy and surgical risks can be difficult using verbal explanations or simple drawings alone. In this randomized study, we compared traditional drawing-based counseling with communication supported by a virtual three-dimensional (3D) thyroid model in patients with newly diagnosed thyroid cancer. We found that patients who used the 3D model had a better understanding of key anatomical structures, such as the recurrent laryngeal nerve and lymph node dissection extent, as well as the mechanisms underlying major postoperative complications, including hoarseness, hypocalcemia, and bleeding. These findings suggest that virtual 3D thyroid models can enhance patient comprehension and support shared decision-making. Incorporating 3D visualization into routine preoperative counseling may represent a simple and effective way to improve patient education in thyroid surgery.

## 1. Introduction

Thyroidectomy is a complex procedure due to the close proximity of the thyroid gland to major arteries, veins, the recurrent laryngeal nerve, the parathyroid glands, the trachea, and the esophagus [1,2]. In recent years, growing public awareness and improved accessibility of thyroid examinations have led to a marked increase in the detection of thyroid cancer and, consequently, in the number of thyroidectomies performed [3,4,5,6]. This trend has created a rising demand for comprehensive perioperative patient education on thyroid surgeries.

With the increasing availability of health information and routine screening, patients are becoming more aware of thyroid cancer and are paying closer attention to surgical options and expected outcomes. Although surgery remains the cornerstone of thyroid cancer treatment, thorough preoperative communication is essential [7,8]. However, explaining the intricate anatomy of the thyroid gland and its surrounding structures during routine clinical practice poses a considerable challenge [9]. Traditional approaches through verbal descriptions or two-dimensional illustrations may not provide sufficient clarity, which can potentially compromise patients’ comprehension and impair effective shared decision-making.

A virtual three-dimensional (3D) thyroid model offers a potential solution, providing an intuitive visual tool that enhances patient understanding and facilitates clinician–patient communication [10,11,12]. While individualized imaging-based 3D-printed models have been shown to improve patient understanding, their widespread use is constrained by high costs and time-consuming production processes [13]. In contrast, virtual 3D thyroid models developed with digital technologies provide several advantages, including lower cost, broader accessibility, and the capacity for dynamic visualization [14,15]. Such virtual models can illustrate the complex anatomy of the thyroid gland and adjacent structures, depict diverse pathologies, highlight potential invasion of critical structures, and demonstrate surgical dissections of the thyroid gland and regional lymph nodes.

The present study evaluates the application of a virtual 3D thyroid model, the Thyroid Navigator app, as a preoperative communication tool between surgeons and patients. By providing comprehensive and individualized information about anatomical structures, surgical procedures, and potential postoperative outcomes, this approach is expected to enhance patient understanding of surgical anatomy and procedures.

## 2. Materials and Methods

### 2.1. Study Design and Participants

**Figure 1:**
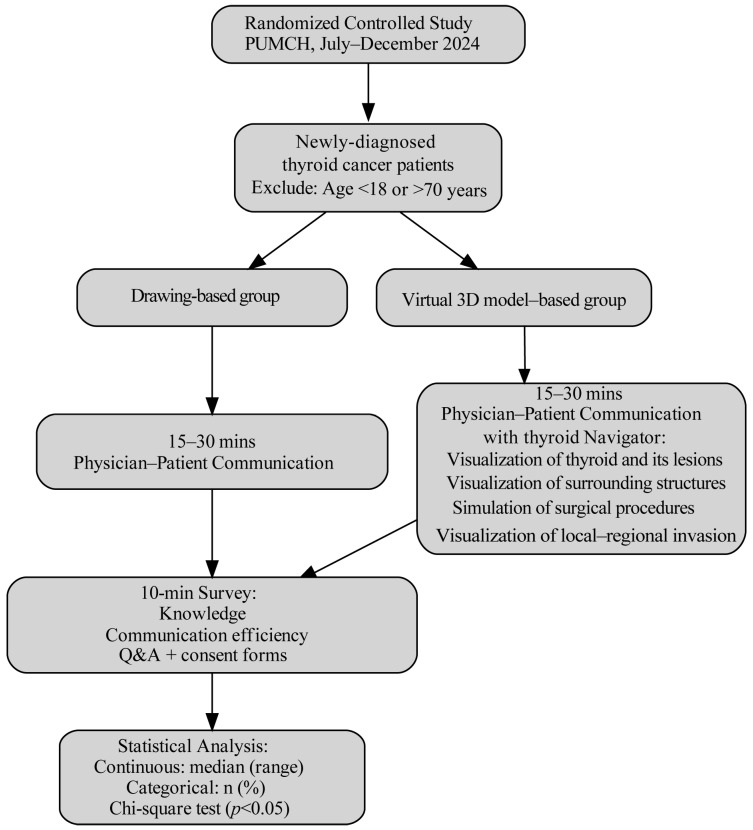
Study flow diagram of the randomized controlled trial. Ninety-four eligible patients with newly-diagnosed thyroid cancer were randomly assigned in a 1:1 ratio to either the schematic drawing-based communication group (*n* = 47) or the virtual 3D model-based communication group (*n* = 47). All patients received standardized preoperative counseling and subsequently completed a structured survey assessing understanding in pertinent anatomy and postoperative complications.

This randomized controlled study was conducted at Peking Union Medical College Hospital between July and December 2024. Consecutive patients with histologically confirmed thyroid cancer on fine-needle aspiration biopsy and no prior history of medically or surgically treated conditions were considered eligible. Patients younger than 18 years or older than 70 years were excluded. A total of 94 eligible patients scheduled for thyroidectomy were recruited and randomly assigned to the control group (drawing-based communication, *n* = 47) or the intervention group (virtual 3D model-based communication, *n* = 47) in a 1:1 ratio. Randomization was performed using a computer-generated sequence. In the control group, communication was conducted using standardized two-dimensional schematic images, which were pre-printed and identical for all patients, rather than being individually hand-drawn by surgeons. In the intervention group, communication was conducted using a virtual three-dimensional thyroid model. Each patient participated in a standardized 15–30 min preoperative communication session with an attending surgeon one day prior to surgery. The study design is illustrated in Figure 1. This study was approved by the Institutional Review Board of Peking Union Medical College Hospital (No. JS3267). All participants provided written informed consent prior to enrollment.

### 2.2. Virtual 3D Thyroid Model

With the increasing adoption of mobile technologies in medicine, medical applications based on 3D reconstruction have gained widespread acceptance. The Thyroid Navigator app was developed to provide an interactive and anatomically accurate 3D model of the thyroid gland and its surrounding structures for surgical education and preoperative counseling. Its main functions include:

(1) Visualization of normal thyroid structures: the left and right lobes, isthmus, and pyramidal lobe.
(2) Visualization of the surrounding structures: major arteries (the aortic arch, brachiocephalic trunk, bilateral common carotid, superior and inferior thyroid arteries), veins (the superior vena cava, subclavian veins, internal jugular veins, superior/middle/inferior thyroid veins), nerves (the vagus, superior laryngeal branches, recurrent laryngeal nerves), the respiratory and digestive tracts (the trachea, larynx, esophagus, hyoid bone, thyroid cartilage, thyrohyoid membrane), and the parathyroid glands.
(3) Demonstration of thyroid and parathyroid pathologies: diffuse goiter, nodular goiters in various locations, and parathyroid tumors.
(4) Simulation of surgical procedures: lobectomy, lobe-isthmectomy + central lymph nodes dissection, total thyroidectomy, total thyroidectomy + central lymph node dissection, and total thyroidectomy + lateral neck lymph nodes dissection.
(5) Visualization of local–regional invasion including recurrent laryngeal nerve involvement, tracheal invasion, and esophageal invasion.
(6) Customized display options: selective removal of blood vessels or skin to enhance anatomical clarity.

In addition to reconstruct the thyroid gland and tumor, the app integrates critical adjacent structures to contextualize surgical risks. Awareness of these anatomical relationships is essential for surgical planning, as cancer invasion of vital structures can have significant clinical consequences. For example, vascular invasion may necessitate ligation of the jugular vein or carotid artery, leading to compromised cerebral blood flow; tracheal invasion may require segmental resection to maintain airway patency; and recurrent laryngeal nerve involvement can cause irreversible vocal cord dysfunction.

### 2.3. Survey and Outcome Measures

Following the preoperative communication session, all patients completed a structured 10 min survey designed to assess both objective understanding and subjective perception of communication effectiveness. Objective understanding was evaluated with these items: (a) lesion location identification, (b) parathyroid gland identification, (c) recurrent laryngeal nerve recognition, (d) extent of lymph node dissection, (e) common postoperative complications. Each response was scored on a three-point scale: 1 = totally incorrect, 2 = partially correct, or 3 = completely correct. During the survey, patients were also encouraged to raise additional questions concerning their surgery and expected postoperative outcomes. The 3-point scale was used to assess the accuracy of the patients’ understanding of complication mechanisms, rather than perceived risk or probability of complication occurrence.

### 2.4. Statistical Analysis

Continuous variables were summarized as medians with interquartile ranges, whereas categorical variables were presented as absolute counts and percentages. Group differences for categorical variables were analyzed using Chi-squared or Fisher’s exact tests, as appropriate, and continuous variables were compared using the Mann–Whitney U test. A two-tailed *p* value of <0.05 was considered statistically significant. All statistical analyses were performed using SPSS version 20.0 for Windows (SPSS Inc., Chicago, IL, USA).

## 3. Results

### 3.1. Virtual 3D Thyroid Visualization App

**Figure 2:**
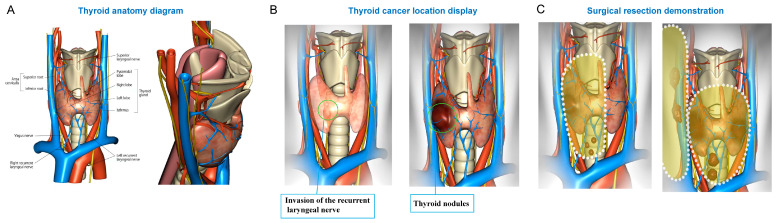
User interface of the virtual 3D thyroid model (Thyroid Navigator app). (**A**) Visualization of normal thyroid anatomy, including the bilateral lobes, isthmus, parathyroid glands, and adjacent structures such as trachea and major vessels. (**B**) Thyroid cancer location display and representation of nerve invasion. (**C**) Simulation of surgical resection.

The Thyroid Navigator app was developed by Kuma Hospital to allow for visualization of thyroid nodules in different locations with possible local invasion and to simulate the effects of surgical resections through variable approaches. The app also incorporates normal thyroid anatomy and surrounding critical structures. The user interface of the virtual 3D thyroid model used in this study is illustrated in Figure 2.

### 3.2. Patient Characteristics

The clinicopathological characteristics of the enrolled patients are summarized in Table 1. A total of 94 patients with newly-diagnosed papillary thyroid carcinoma were included, with 47 patients in the drawing-based communication group and 47 in the virtual 3D model-based group. The mean age was 42.021 ± 13.699 years in the drawing-based group and 42.702 ± 11.705 years in the virtual 3D model-based group (*p* = 0.796). Most of the patients were females (76 females vs. 24 males). The median tumor size did not differ significantly between groups (*p* = 0.383). The distribution of surgical procedures was comparable between groups (*p* = 0.233). No postoperative complications related to nerve injury, bleeding, or hypocalcemia were observed in this cohort. Patients were typically discharged on postoperative day 2 or 3, once the drainage was clear and <20 mL/24 h.

### 3.3. Patient Understanding in Pertinent Anatomy and Postoperative Complications

**Figure 3:**
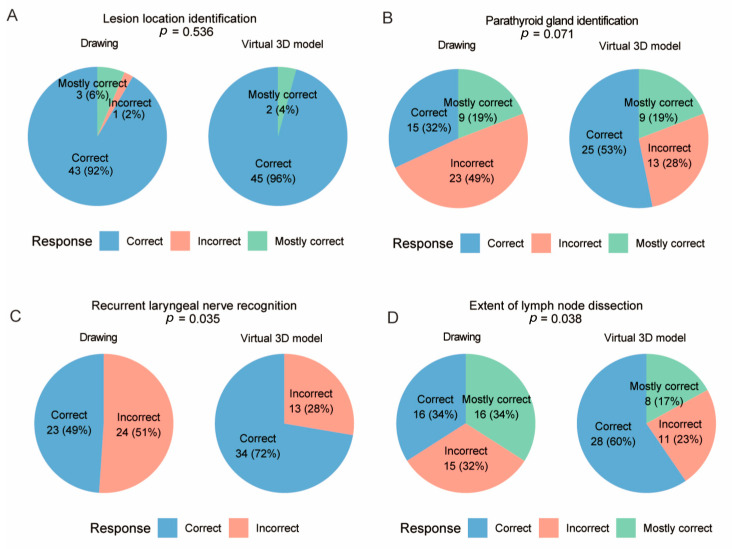
Accuracy in anatomical recognition. Correct response rates in the drawing-based and virtual 3D model-based groups for identification of the lesion location (**A**), parathyroid glands (**B**), recurrent laryngeal nerve (**C**), and extent of lymph node dissection (**D**).

**Figure 4:**
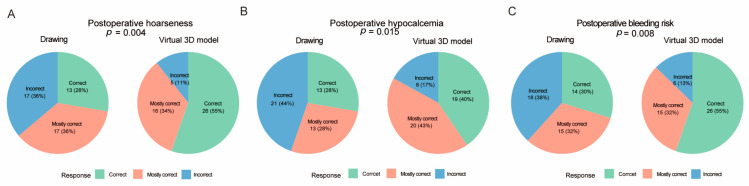
Understanding in causes of postoperative complications. Correct response rates in the drawing-based and virtual 3D model-based groups for the identification of the causes of postoperative hoarseness (**A**), hypocalcemia (**B**), and bleeding (**C**).

The correct response rate for lesion localization was comparable between the two groups (*p* = 0.536, Figure 3A). An increase trend in the correct response rate for parathyroid gland identification was observed in the virtual 3D model-based group, although statistical significance was not reached (*p* = 0.071, Figure 3B). In contrast, patients in the virtual 3D model-based group demonstrated significantly higher accuracy in identification of the recurrent laryngeal nerve (*p* = 0.035, Figure 3C), and extent of lymph node dissection (*p* = 0.038, Figure 3D), which suggests improvement in patients’ anatomical understanding. Moreover, the accuracy in identifying the causes of major postoperative complications—including hoarseness (caused by recurrent laryngeal nerve injury), hypocalcemia (parathyroid gland impairment), and bleeding (inadequate hemostasis)—was significantly higher in the virtual 3D model-based group than in the drawing-based group (*p* < 0.05, Figure 4). This indicates that the virtual 3D model also enhances patients’ comprehension of potential postoperative complications. To further quantify the strength of association between communication method and item-level response distributions, effect sizes (Cramer’s V) were calculated and *p*-values were adjusted for multiple comparisons using the Holm method (Supplementary Table S1).

### 3.4. The Association Between Educational Level and Correct Responses

Further analysis showed that patients with a junior high school education or below performed comparably to those with higher education on relatively straightforward items such as lesion localization (90.0% vs. 92.7% vs. 97.0%, *p* = 0.731, Table 2). However, for more conceptually complex topics, their correct response rates were substantially lower, including recurrent laryngeal nerve recognition (35.0% vs. 70.7% vs. 63.6%, *p* = 0.027) and extent of lymph node dissection (25.0% vs. 51.2% vs. 54.5%, *p* = 0.143). Similarly, understanding of postoperative complications showed a clear educational gradient, with lower correct rates in the junior high school education or below group for postoperative hoarseness (30.0%, *p* = 0.042), hypocalcemia (15.0%, *p* = 0.010), and bleeding risk (20.0%, *p* = 0.002). In contrast, correct response rates were generally comparable between the high school/technical school and college-or-above groups across most items.

## 4. Discussion

Previous studies have demonstrated that 3D visualization tools, particularly customized 3D-printed models, can enhance medical education and patient communication. For instance, 3D-printed phantoms have been applied in conventional otosurgical training to support accurate surgical planning and resident education [16]. A synthetic simulator combining silicone and 3D-printed components has been used for orbitozygomatic fracture training, providing a cost-effective tool with strong structural validity [17]. Similarly, a highly anatomically accurate 3D model has been successfully employed to help residents learn and improve their performance in percutaneous transhepatic bile duct drainage [18]. In the context of thyroid cancer, a CT-based 3D thyroid cancer phantom has been shown to improve patient education through facilitating their understanding of the disease [19].

Despite these benefits, widespread adoption of individualized 3D-printed models in routine thyroid surgery remains limited [20]. Their production is both costly and time-consuming, rendering their routine use impractical for the large number of thyroidectomies performed daily [21]. Furthermore, the static nature of 3D-printed phantoms restricts their educational value, as they cannot be manipulated to simulate surgical dissections or permit selective removal of superficial layers to reveal underlying structures [22]. These limitations underscore the need for more flexible and accessible alternatives.

In this context, the present study evaluated a dynamic virtual 3D thyroid model, the Thyroid Navigator app, as a novel tool for preoperative patient counseling. Compared with 3D-printed phantoms, the virtual model provides detailed information on relevant anatomy, greater interactivity, and dynamic visualization capabilities. It allows surgeons to toggle between superficial and deep structures and to simulate different surgical approaches, thereby enabling a more comprehensive and intuitive explanation of the surgical procedures. These features distinguish the virtual model not only from 3D-printed phantoms but also from traditional atlases or hand-drawn illustrations, which lack comparable spatial resolution [23].

In our study, we observed significant advantages of the virtual 3D thyroid model in improving patients’ understanding across multiple domains. For instance, the rate of incorrect responses in parathyroid gland identification nearly halved in the 3D model-based group compared with the drawing-based group, even though the overall performance did not reach statistical significance. Notably, the recurrent laryngeal nerve, which was the most challenging concept in the conventional group with the highest error rate, was correctly identified by the majority of the participants in the virtual 3D group. Similarly, understanding of the postoperative risks, including all three elements surveyed, was significantly enhanced. These findings highlight that the virtual model not only improved patients’ anatomical awareness but also deepened their grasp of surgical risks, thereby promoting more informed preoperative decision-making. It should be emphasized that the endpoints of this study reflect patients’ understanding of surgical anatomy and complication mechanisms, rather than actual surgical outcomes or complication rates. Beyond patient counseling, these results suggest that such virtual tools could also support surgical education by reinforcing critical anatomical recognition in inexperienced clinicians.

This study has several limitations. First, the 3D model used was not patient-specific. It was based on a standardized anatomical atlas rather than CT-derived reconstruction for individual patients. This design choice was made to prioritize accessibility and routine clinical applicability for patient communication rather than individualized surgical navigation. Currently, software capable of generating patient-specific virtual thyroid models is not widely available. Future advances in digital reconstruction may enable personalized models tailored to patients’ imaging data, further enhancing individualized preoperative counseling. Second, this was a single-center study with a moderate sample size and included only patients with papillary thyroid carcinoma, which may limit the generalizability of the findings to other thyroid diseases or clinical settings. Multicenter studies with broader disease spectra are warranted to validate and extend these results. Finally, due to the nature of the intervention, complete blinding of patients and surgeons was not feasible, which may introduce potential information bias. In addition, the questionnaire used in this study was study-specific and designed to assess immediate post-counseling understanding; it has not undergone formal psychometric validation, and reliability or internal consistency metrics were not evaluated. However, outcome assessment was based on standardized patient-completed questionnaires with predefined scoring criteria, helping to mitigate this limitation. Long-term outcomes, such as postoperative satisfaction, decision regret, anxiety, and adherence to follow-up care, were not assessed and should be explored in future research.

## 5. Conclusions

This randomized controlled study showed that the Thyroid Navigator virtual 3D model is an effective adjunct to conventional preoperative communication in thyroid surgery. The use of this tool enhanced patients’ understanding of thyroid anatomy, surgical procedures, and potential complications, thereby facilitating informed decision-making. Moreover, the model had educational value for junior surgeons. Although not yet patient-specific, virtual 3D models offer a practical and interactive approach to improving communication in routine thyroid surgical practice, with potential for further refinement as technology.

## Figures and Tables

**Table 1:** Clinicopathological parameters of patients in two groups.

	Drawing-Based Group (*n* = 47)	Virtual 3D Model-Based Group (*n* = 47)	*p* Value
Age (years)	42.021 ± 13.699	42.702 ± 11.705	0.796
Sex			0.344
Female	40	36	
Male	10	14	
Tumor size (cm)	0.883 ± 0.609	0.769 ± 0.651	0.383
Extent of surgery			0.233
Lobectomy + unilateral central neck dissection	11	16	
Total thyroidectomy + unilateral central neck dissection	12	17	
Total thyroidectomy + bilateral central neck dissection	13	8	
Total thyroidectomy + central neck dissection + unilateral neck dissection	9	6	
Total thyroidectomy + central neck dissection + bilateral neck dissection	2	0	
Education level			0.146
Junior high school or below	9	11	
High school/technical school	17	24	
College or above	21	12	

**Table 2:** Association between education level and correct responses across assessment items.

Assessment Item	Junior High or Below	High School/Technical	College or Above	*p* Value
Lesion localization	18 (90.0%)	38 (92.7%)	32 (97.0%)	0.731
Parathyroid gland identification	8 (40.0%)	18 (43.9%)	14 (42.4%)	0.221
Recurrent laryngeal nerve recognition	7 (35.0%)	29 (70.7%)	21 (63.6%)	0.027
Extent of lymph node dissection	5 (25.0%)	21 (51.2%)	18 (54.5%)	0.143
Postoperative hoarseness	6 (30.0%)	19 (46.3%)	14 (42.4%)	0.042
Postoperative hypocalcemia	3 (15.0%)	16 (39.0%)	13 (39.4%)	0.01
Postoperative bleeding risk	4 (20.0%)	21 (51.2%)	15 (45.5%)	0.002

## Data Availability

All data generated or analyzed during this study are included in this article. The original data will be available from corresponding authors on reasonable request.

## Supplementary Materials

The following supporting information can be downloaded at: https://www.mdpi.com/article/10.3390/cancers18020241/s1, Table S1: Effect sizes and adjusted *p*-values for item-level comparisons between drawing-based and 3D model-based communication.
